# Supplementary material for: Community perspectives on cardiovascular disease control in rural Ghana: A qualitative study
Source: PLoS One. 2023 Jan 20;18(1):e0280358. doi: 10.1371/journal.pone.0280358 (PMC9858357; doi:10.1371/journal.pone.0280358)
Supplement: S1 Appendix — (PDF) [file pone.0280358.s001.pdf]

Interview Guide, Community Member

This survey is designed to assess your views about heart disease in the community. It is confidential - although we will review your results with our research team and the CHPS staff, this review will be anonymous and will not identify you in any way. You can stop at any time during this interview.

1. What have you heard before about heart disease?

2. Do you know anyone with heart disease?

3. When people are sick with heart disease, what kinds of symptoms/problems do they have?

4. Do you think CHPS providers good care for heart disease? Why or why not?

5. How could CHPS provider better care for heart disease?

6. What are the best ways to prevent heart disease?

7. What kinds of food are healthiest for preventing heart disease?

8. What kinds of medicines are used for treating heart disease?
