# Supplementary material for: Community perspectives on cardiovascular disease control in rural Ghana: A qualitative study
Source: PLoS One. 2023 Jan 20;18(1):e0280358. doi: 10.1371/journal.pone.0280358 (PMC9858357; doi:10.1371/journal.pone.0280358)
Supplement: S1 File — (PDF) [file pone.0280358.s002.pdf]

Inclusivity in global research

PLOS' policy on inclusivity in global research aims to improve transparency in the reporting of research performed outside of researchers' own country or community and ensures that PLOS publications reporting global research adhere to high standards for research ethics and authorship. Authors of relevant research articles may be asked to complete the questionnaire below, which outlines ethical, cultural, and scientific considerations specific to inclusivity in global research. This questionnaire may be requested when researchers have travelled to a different country to conduct research, if research uses samples collected in another country, research with Indigenous populations or their lands, or if research is on cultural artefacts. Researchers travelling to another country solely to use laboratory equipment will not normally be required to complete the questionnaire. However, the questionnaire can be requested at the journal's discretion for any submission – if you have been requested to complete this questionnaire by the PLOS journal you submitted to, please do so.

Please complete the questionnaire below and include this as a Supporting Information file with your manuscript. Note that if your paper is accepted for publication, this checklist will be published with your article in the supporting information files. Please ensure that you reference the checklist in the main body of your manuscript. We suggest adding a subsection 'Inclusivity in global research' to your Methods section and adding the following sentence: "Additional information regarding the ethical, cultural, and scientific considerations specific to inclusivity in global research is included in the Supporting Information (SX Checklist)"

The questions have been designed to be applicable to a wide range of study types, and there are subsections for both human subjects research and non-human subjects research. If any of the questions are not relevant to your research please mark them as "N/A" as appropriate.

Ethical considerations, permits and authorship

This section is applicable to all research types.

Provide details as to who granted permissions and/or consent for the study to take place in the Methods section of your manuscript. This should include the names of **all** ethics boards, governmental organizations, community leaders or other bodies that provided approval for the study. If individuals provided approval refer to these people by their role or title but do not list their name(s).

Reported on page number: 5, specifically under "IRB Approval." We received approval from the ethics boards of both the Navrongo Health Research Centre in Navrongo, Ghana and the Icahn School of Medicine at Mount Sinai in New York, USA.

If there were any deviations from the study protocol after approval was obtained please provide details of these changes in the Methods section of your manuscript.

We report no deviations in our study protocol following the ethical approvals above.

Did this study involve local collaborators that are residents of the country where the research was conducted or members of the community studied? If you do not have any authors from said communities, please provide an explanation for this below.

The study involved multiple collaborators from the Navrongo Health Research Centre, three of who are co-authors on this manuscript: Dr. Raymond Aborigo, Mr. Denis Awuni, and Dr. Abraham Oduro. These three scholars are both resident in the Navrongo community where the research occurred and expert in its culture and language.

Everyone listed as an author should meet PLOS' criteria for authorship and all individuals who meet these criteria should be included in the author byline, rather than the acknowledgements. Authorship criteria is based on the International Committee of Medical Journal Editors (ICMJE) Uniform Requirements for Manuscripts Submitted to Biomedical Journals - for further information please see here: https://journals.plos.org/plosone/s/authorship.

Human subjects research (e.g. health research, medical research, cross-cultural psychology)

Did you obtain written informed consent from a representative of the local community or region before the research took place? How did you establish who speaks for the community? Details of written informed consent obtained from study participants should be reported separately in the Methods section of your manuscript.

The institutional review board (IRB) of the Navrongo Health Research Centre, from whom we obtained written informed consent before beginning this research, comprises community leaders from the Navrongo communities where the research took place. We recruited members of these communities, as detailed in the Methods section of the manuscript, as a random sampling of the community as a whole rather than as leaders to speak on the community's collective behalf.

How did members of the local community provide input on the aims of the research investigation, its methodology, and its anticipated outcome(s)?

This research and related work was preceded by (and followed by) a series of in-person meetings with sub-district, district, and regional health leaders as well as elected community leaders. The goal was to make community members and health providers aware of who is engaged in this research; the aim to work with the community to improve local chronic disease care access, and the expected next step of piloting a community intervention to this effect. More specifically, the goal of this research manuscript *itself* was to elicit community input on our intervention, i.e. to ask community members how best to locally screen for and treat cardiovascular conditions such as hypertension.

When engaging with the local community, how did you ensure that the informed consent documents and other materials could be understood by local stakeholders?

All informed consent discussions were conducted by research staff from the Navrongo Health Research Centre fluent in the local Kasem and/or Nankam languages in addition to English. As detailed in the Methods section of the manuscript, these conversations were conducted orally in the preferred language of the respondent to ensure complete and informed comprehension.

Will the findings of the research be made available in an understandable format to stakeholders in the community where the study was conducted (e.g. via a presentation, summary report, copies of publications, etc.)? Please provide details of how this will be achieved.

Yes – we have already shared preliminary findings from our research to date orally with community leaders and health program directors representing seven communities in the region over multiple meetings since 2019. Separately, we plan to directly inform community members of our findings from this research manuscript, and others, in the form of community gatherings known as durbars in early 2023. This work will support enrollment in a chronic disease intervention now in its pilot phase that we will scale up as a direct result of our findings.

Non-human subjects research using specimens/ animals collected as part of the study, or those housed in archival collections. Examples include archaeology, paleontology, botany and zoology.

Did the permission you obtained from a local authority to perform the study include an agreement on access to outputs and benefit sharing? This may include procedures to enable fair distribution of the benefits and resources arising from the research performed. Please include any details of Prior Informed Consent and Benefit Sharing Agreements obtained. These may be required by field-specific regulations, for example the Convention on Biological Diversity (CBD) and the associated Nagoya Protocol.

Our research involved only interviews and focus groups, and no specimens of any kind were collected.

If the material used in your study was imported, please A) provide the year it was imported and B) indicate whether permits were obtained to import/export the materials used, C) provide details of any permits obtained. If this information is not available, please indicate this.

No specimens or materials were collected or imported. Our data consists only of audio transcripts, obtained with informed consent and stored securely at the Navrongo Health Research Centre as per institutional review board protocols.

If you used archival specimens, please state how the material used in your study was acquired by the institute it is held in and provide details of any permits obtained for the original excavations/ sample collection. If this information is not available, please indicate this.

We collected no such materials or specimens.

How was the potential cultural significance of the materials collected in your study to local communities considered in your research design? Were Indigenous peoples and/or local researchers and institutions

involved with archaeological excavations / collection of specimens? If so, please provide a description of their involvement.

We collected no such materials or specimens.

If your manuscript includes photographs of human remains please indicate whether authors obtained permission from descendants or affiliated cultural communities to do so.

Our manuscript has no such photographs.
